# An online survey on coping methods for genitourinary syndrome of menopause, including vulvovaginal atrophy, among Japanese women and their satisfaction levels

**DOI:** 10.1186/s12905-023-02439-4

**Published:** 2023-05-24

**Authors:** Hiroaki Ohta, Mariko Hatta, Kuniaki Ota, Remi Yoshikata, Stefano Salvatore

**Affiliations:** 1 Department of Obstetrics and Gynecology, Kawasaki Medical School, 2-6-1 Nakasange, Kita-Ku, 700-8505 Okayama, Japan; 2 Juno-Vesta Clinic Hatta, Chiba, Japan; 3 Department of Obstetrics and Gynecology, Tokyo Rosai Hospital, Tokyo, Japan; 4 Hamasite Clinic, Tokyo, Japan; 5 Obstetrics and Gynecology Unit, Vita-Salute San Raffaele University, IRCCS San Raffaele Hospital, Urogynecology Unit, Milan, Italy

**Keywords:** Coping methods, Genitourinary syndrome of menopause, Japanese women, Nationwide online survey, Treatment satisfaction, Vulvovaginal atrophy

## Abstract

**Background:**

This study aimed to explore the current situation and existing issues regarding the management of vulvovaginal atrophy (VVA) or the genitourinary syndrome of menopause (GSM). A nationwide web-based questionnaire survey was conducted among 1,031 Japanese women aged 40 years or older.

**Materials and methods:**

Eligible women were asked to complete a questionnaire about how they dealt with their symptoms and how satisfied they were with their coping methods.

**Results:**

Of those highly conscious of their GSM symptoms (*n* = 208; 20.2%), 158 had sought medical consultation (15.3%), with only 15 currently continuing to seek consultation (11.5%). Of the specialties consulted, gynecology was the most frequently consulted (55%). Furthermore, those unwilling to seek medical consultation despite their symptoms accounted for the greatest proportion (*n* = 359; 34.8%), with 42 (23.9%) having never sought consultation. Topical agents, e.g., steroid hormone ointments/creams, were the most frequent treatments provided by the clinics (*n* = 71; 40.3%), followed by oral and vaginal estrogens (*n* = 27; 15.5%), suggesting that estrogen therapy was not the first choice of treatment at the clinics. While 65% of patients treated at the clinics reported satisfaction with the treatments, this was inconsistent with the fact that many were reported to have remained untreated and very few continued with treatment.

**Conclusions:**

Survey results suggest that GSM, including VVA, remains underdiagnosed and undertreated in Japan. Medical professionals should deepen their understanding of GSM and raise their level of care to select the appropriate treatment for the condition.

## Background

In 2014, “genitourinary syndrome of menopause” (GSM) was chosen by the International Society for the Study of Women’s Sexual Health (ISSWSH) and the North American Menopause Society (NAMS) to describe the genital symptoms associated with menopause as an alternative to the term “vulvovaginal atrophy” (VVA), which does not adequately describe the wide range of symptoms associated with menopause, including lower urinary tract symptoms (LUTS) [1, 2]; moreover, this term has been sanctioned by other relevant professional societies across the world. In this regard, our previous survey of Japanese women in their 40 s or older [3] showed that genital and lower urinary tract symptoms are more frequently found together than in isolation. Despite the recognized need for effective treatment for voiding dysfunction in middle-aged and elderly patients presenting to gynecology clinics in Japan, the continued use of the term “VVA” in gynecology in Japan is likely to mean that the urinary tract symptoms may often go unheeded or untreated [4].

In contrast, the term GSM has been adopted in several Western countries as a concept to address the symptoms associated with menopause due to a decline in the sex hormones, such as estrogen, testosterone, and dehydroepiandrosterone (DHEA), which affect not only the genital organs but also the lower urinary tract, thus extending to some sexual function-related symptoms [5]. Thus, the concept of GSM needs to be made clear and more accessible to healthcare providers in Japan to facilitate more comprehensive care for GSM in the clinical setting.

In our previous web-based survey of over 10,000 Japanese women in their 40 s and older [3], approximately 45% reported both genital and urinary tract symptoms, implying that GSM affects not only postmenopausal women but also pre- or perimenopausal women if these are caused due to GSM. Thus, given the possibility that declining estrogen may not only lead to loss of bone and deterioration of lipid metabolism but also to VVA, thereby affecting a wide range of organs [6], symptoms of VVA/GSM may also be found in perimenopausal women or those in the menopause transition, which is supported by a recent Italian proposal for dealing with VVA according to three life stages before and after menopause [7].

Furthermore, while women with symptoms of VVA/GSM may choose to seek professional help, many may be hesitant to seek medical consultation about these symptoms, which may become chronic or progressive with aging and cause dyspareunia or incontinence in postmenopausal women, thus adversely affecting their quality of life (QOL) and sexual function [8–13]. VVA/GSM represents a key disease concept in achieving improved QOL or health in middle-aged and elderly women in developed countries characterized by the conspicuous societal aging,

Against this background, of the 10,000 premenopausal and postmenopausal women who had participated in an earlier online survey, those who reported symptoms likely due to VVA/GSM were further asked to respond to six questions in a questionnaire designed to investigate how they were coping with their symptoms and how satisfied they were with their coping methods, to provide insight into the challenges in the clinical management of VVA/GSM.

The purpose of this study was to investigate the level of satisfaction with the effects of treatment based on the type of treatment provided, and whether the symptoms the patient had before the visit actually improved as a result of the treatment.

## Materials and methods

A nationwide, large-scale online questionnaire survey—provided by the Internet survey company Macromill Carenet Inc.—was conducted among women in their 40 s or older using questions about the symptoms of GSM from February 2–4, 2017 [3]. Of the 10,000 individuals surveyed, 4488 (44.9%) experienced genital or urinary tract symptoms. Of these, a total of 1,031 women aged between 40 and 90 years reported symptoms likely due to GSM (40 s, *n* = 258; 50 s, *n* = 412; 60 s or older, *n* = 361) and were further followed up by e-mail and asked to answer the six questions listed in Table 1.

**Table 1:** Current questionnaire survey (*n* = 1,031)

Q1. Were you aware that the vulva, vagina, and urinary tract undergo age-related changes?
(A) Yes/No, I was not aware of this
Q2. If you answered, I am “very worried,” “worried,” or “somewhat worried” about vulvar, vaginal, or urinary tract symptoms, are you doing something to manage these symptoms?
(A) I am receiving consultation
I have received consultation in the past (but I am currently receiving none)
I am managing the symptoms with over-the-counter medicines
I am thinking about what to do but have not actually done anything
I want to do something but do not know what to do
I feel that I do not require treatment
Q3. If you answered, “I am receiving/have received consultation” in Q2, which specialty are you consulting/have you consulted?
(A) Gynecology/Urology/Dermatology/Internal Medicine/Family Medicine/Other
Q4. If you answered, “I am currently receiving/have received consultation” in Q2, what kind of treatment have you received?
(A) Ointment or cream/Lubricating jelly/Local estrogen therapy (vaginal suppository)/ Systemic hormone replacement therapy (HRT)/Other/I had an examination but have not received any treatment
Q5. If you answered, “I am receiving/have received consultation” in Q2, how satisfied are/were you with your treatment?
(A)Not satisfied at all/Not satisfied/Not very satisfied/Somewhat satisfied/Satisfied
Q6. If you answered, “I have received consultation in the past (but I am currently receiving none) in Q2, please give reasons for currently receiving no consultation currently
(A) I find the condition bearable
It is not bad enough to require consultation
It is an age-related problem and therefore inevitable
I am dealing with it on my own
I find it difficult to seek consultation
It may incur unnecessary costs
I don’t know what kind of treatment will be given
I feel anxious about the treatment to be given
I am afraid no coping method will be found for the symptom even after examination
Other

The study protocol (“A web-based questionnaire survey on genitourinary syndrome of menopause [GSM]”) was submitted to and approved (approval number: 5486–00) as eligible for implementation/continuation by the institutional review board (IRB) of Kawasaki Medical School, Okayama, Japan, while having been judged as exempt from IRB review. Informed consent was obtained from all participants prior to study participation.

## Results

The characteristics of the 1,031 respondents in this study are shown in Table 2 (mean age [range]: 55.6 ± 9.3 years [40–86 years]). Of these, 72.9% of respondents were married, 74.3% had children, and 30.4% were sexually active. Of these respondents, 597 (57.9%) reported going out of the home as often as 3 to 6 days per week, suggesting that the respondents represented a normally active population.

**Table 2:** Background characteristics of survey respondents (*n* = 1,031)

Age group	N	Mean age (years)	Married (%)	W/Children (%)	Partnered (%)	Sexually active (%)
40–49	258	44.1 ± 2.8	68.6	60.5	77.1	45.7
50–59	412	53.5 ± 2.6	73.8	73.1	79.1	30.8
≥ 60	361	66.1 ± 4.7	75.1	85.6	77.0	18.8
Total	1031	55.6 ± 9.3	72.9	74.3	77.9	30.4

**Fig. 1:**
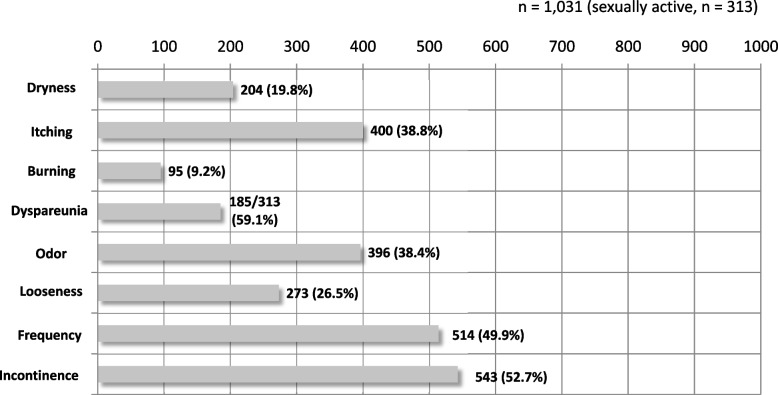
Genitourinary symptoms reported by the respondents (*n* = 1,031). Of the genitourinary tract symptoms, urinary symptoms are commonly reported. However, dyspareunia is the most reported of all genitourinary symptoms

Of the eight genital or urinary tract symptoms evaluated in the survey (Fig. 1), “urine leakage” (incontinence) was the most common among the 1,031 respondents, with 543 (52.7%) reporting the presence of this problem, followed by 514 (49.9%) respondents experiencing “frequent urination” (urinary frequency). Thus, urinary tract symptoms were reported more frequently than genital symptoms. Of the 1,031 respondents, 313 reported being sexually active ( +); again, of these, 185 (59.1%) reported experiencing coital pain (or dyspareunia), suggesting that coital pain was the most common symptom among these women. Furthermore, 295 (28.6%) and 282 (27.4%) women reported experiencing either genital or urinary tract symptoms, respectively, and 454 (44.0%) reported experiencing both. This number was more than 1.5 times greater than the number of women who reported genital or urinary symptoms alone.

Sexually active women reported experiencing more genital symptoms (34.5%) than urinary tract symptoms (20.1%), whereas sexually inactive women were likely to report more urinary tract symptoms (30.5%) than genital symptoms (26.0%), in agreement with the survey results reported earlier [3].

**Fig. 2:**
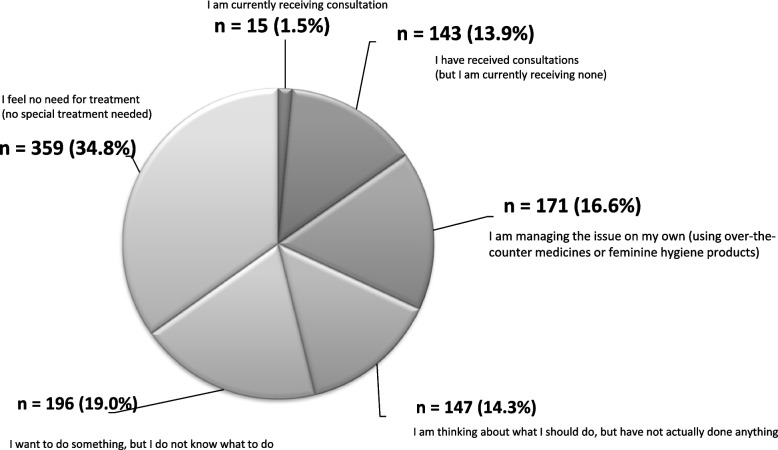
Coping methods for genitourinary symptoms (*n* = 1,031). Perhaps due to differences in the perceived severity of their symptoms or knowledge of coping methods, questionnaire responses vary widely, suggesting general bewilderment

Only 158 respondents (15.3%) reported having received or are currently receiving consultation to cope with these symptoms, despite the fact that a high proportion of respondents (681; 66.1%) were aware that these symptoms were age-related changes. However, those who did not currently feel the need for care (359; 34.8%) accounted for the largest proportion of the respondents, followed by those who did not know how to cope with their symptoms (196; 19.0%), those managing their symptoms on their own with over-the-counter medicines or feminine hygiene products (171; 16.6%), and those who felt the need for care but were not addressing the need (147; 14.3%) (Fig. 2). Furthermore, of the specialties consulted by the respondents, gynecology (55%) was the most common, followed by urology (27%), internal medicine (9%), dermatology (5%), and family medicine (3%).

The primary reasons given (1,720; multiple answers allowed) by the 873 respondents who reported symptoms for not seeking medical consultation were, in decreasing frequency, as follows: that they found the symptoms bearable (501; 57.4%); that the symptoms were not bad enough to seek medical consultation (281; 32.3%); that the symptoms were assumed to be age-related and therefore inevitable (242; 28.5%); and that they found it difficult to seek medical consultation about their symptoms (188; 21.5%) (Table 3).

**Table 3:** Hospital-provided treatments (*n* = 176): The majority report receiving (having received) ointments/creams or hormone therapy (more vaginal suppositories than oral agents)

Treatment	134 (75%)
Ointments and creams	71 (40.3%)
Lubricating jelly	4 (2.3%)
Local estrogen treatment (vaginal suppository)	15 (8.5%)
Systematic hormone replacement therapy (HRT)	12 (6.8%)
Other	32 (18.2%)
No treatment	42 (23.9%)

**Fig. 3:**
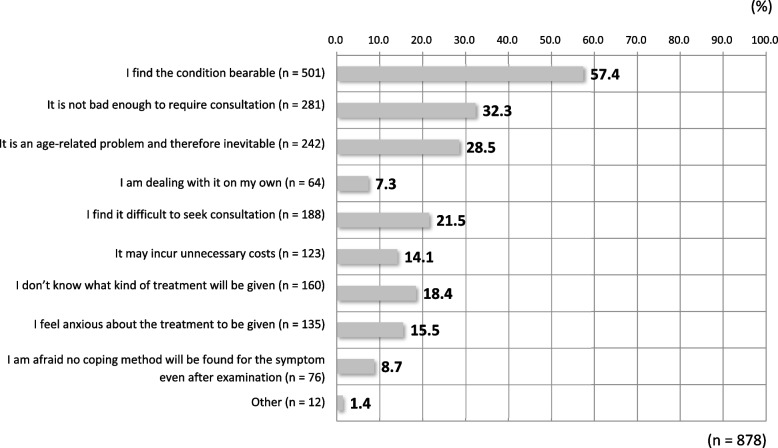
Reasons for not seeking medical consultation (*n* = 873) (multiple answers allowed). These respondents do not seek medical consultation on their own judgment or for no good reason

Among the 158 individuals who reported receiving treatment or having done so in the past, the most common types of treatment received (176; multiple answers allowed) were “steroid hormone ointments or creams” (71; 40.3%), followed by “no treatment” even after consultation (42; 23.9%), “local estrogen therapy” (15; 8.5%), “systemic hormone replacement therapy” (12; 6.8%), and “lubricating jelly” (4; 2.3%) (Table 4). Of these same patients, 116 reported having been treated for the symptoms (multiple answers allowed); of these, 67 (57.8%) reported satisfaction with the treatments, with 30 (25.9%) and 37 (31.9%) found to be very satisfied and somewhat satisfied, respectively (Fig. 3). However, as many as 42 respondents (23.9%) reported receiving no treatment despite seeking consultation. Again, of the 158 respondents who reported having received consultations in the past or receiving consultation at healthcare facilities, as few as 15 (1.5%) continued to receive consultation.

**Table 4:** A quarter and one-third of respondents report being satisfied and somewhat satisfied, respectively, but another quarter report being not very satisfied, indicating suboptimal levels of satisfaction among the respondents

Levels of satisfaction with hospital-provided treatments for genitourinary symptoms (*n* = 116)	
Very satisfied	8 (6.9%)
Satisfied	30 (25.9%)
Somewhat satisfied	37 (31.9%)
Not very satisfied	29 (25.0%)
Not satisfied	7 (6.0%)
Not satisfied at all	5 (4.3%)

## Discussion

This web-based survey of more than 1,000 Japanese women in their 40 s or older who reported symptoms of VVA/GSM demonstrated that these peri- and postmenopausal women tended to leave their symptoms untreated as they considered them to be age-related and therefore inevitable, or they managed their symptoms with non-hormonal treatments such as over-the-counter medicines or self-care products. In addition, those who reported having received or receiving consultation at gynecology and urology clinics accounted for more than 80% of all women who reported having done so or doing so, while only 1.5% of these women continued receiving consultation and accounted for less than 10% of all who reported having done so or doing so (15.4%). This was explained by the fact that topical steroid preparations or low-potency vaginal estriol tablets represent the mainstay of treatment at most healthcare facilities and that oral estrogen was not being proactively used due to its associated safety concerns, such as breast cancer risk.

While treatments available overseas for these symptoms are abound and include estrogen creams [14, 15], vaginal estrogen tablets [16, 17], vaginal rings [18], and vaginal DHEA tablets [19, 20], all of which are currently approved in the US and Canada as effective and safe for clinical use, as well as the oral selective estrogen receptor modulator (SERM), ospemifene [21–23], which is approved for use only in the US, these options remain unapproved for clinical use in Japan, thus likely contributing to the undertreatment of GSM, including VVA, in Japan. Given the presence of the Pharmaceuticals and Medical Devices Agency (PMDA) in Japan as the world’s unique regulatory agency responsible for protection from health hazards, regulatory reviews of drugs and devices, and policy responses to safety concerns, even after considering the small number of patients actually seeking medical consultation about the condition, this points to the failure of the specialties involved to promote the adoption of new treatment options for GSM after due consideration as an issue of great interest in the management of GSM. This is particularly worth noting, given that the undertreatment of GSM due to the unavailability of sufficient treatment options may result in a vicious cycle leading to practitioners’ reduced willingness to diagnose GSM, which may, in turn, directly result in the deleterious undertreatment of the condition. In this context, while the incidence of GSM has been underestimated even in Western countries that are at the forefront of GSM care [8, 23–25], it is of note that GSM has recently been reported in 84% of 900 Australian women at 6 years after menopause [26]. Again, unlike vasomotor symptoms, which are also due to declining estrogen levels in women and have been shown to attenuate with time, GSM is a highly prevalent and progressive syndrome that is less likely to improve without appropriate treatment. Thus, it remains an issue of common interest to the world at large that middle-aged or elderly women who come to seek treatment for their VVA/GSM symptoms have remained a small minority [27, 28]. Indeed, a survey conducted 10 years ago on 1,858 postmenopausal American women with VVA symptoms showed that 50% of these women had never received treatment for their symptoms  [28]. This may not be true for present-day American women, but it may be true for present-day Japanese women, given our current survey results.

In this regard, it appears that the unwillingness on the part of healthcare providers to discuss VVA/GSM symptoms with affected women or their concerns over the use of hormonal therapy due to associated safety issues may have proved a limiting factor for the widespread assessment or treatment of VVA/GSM [29, 30]. However, GSM has been shown to adversely affect the sexual health and quality of life (QOL) of affected women [31]. Furthermore, even sexually inactive women are shown to experience bothersome symptoms due to GSM, which adversely affect their activities of daily living [32].

At present, issues are numerous in the management of GSM. Again, primarily, our current survey results may point to the need to promote awareness among affected women or those at risk so that VVA and GSM symptoms are amenable to improvement through appropriate treatment. However, of note, the “vaginal taboo” remains an issue among these women, affecting their decision to seek care for their symptoms, leading to an underestimation of the incidence of VVA and GSM, as well as to their associated symptoms becoming increasingly less localized/more widespread, chronic, and progressive. This leads to a significant health hazard, compromising not only the physical and mental health but also the sexual health of affected women due to the deterioration of their QOL. Notably, since non-communicable diseases (NCDs) have now been listed among the diseases that represent future health risks, with the implementation of measures for NCDs highlighted as an area of current interest by the World Health Organization (WHO) [33], osteoporosis and dementia are drawing renewed attention as NCDs of interest among middle-aged and elderly women. In this regard, it is worth noting that GSM represents another NCD of more specific and greater interest to these women than osteoporosis and dementia and that GSM as an NCD requires preemptive therapy before it becomes established. This is despite the unwillingness of affected women to seek consultation or treatment and their inability to avoid the consequences of GSM progressively becoming a serious health risk. Again, while GSM is classified uniformly into mild, moderate, and severe categories at present, given its progressive nature, it appears necessary that GSM be staged from a more objective, multilateral perspective and an appropriate treatment policy be proposed to address its stage.

This web-based survey had several limitations. First, the participants of this survey provided their consent through Macromill Carenet Inc.; therefore, the survey results were limited to those from respondents who gave consent through this company and the validity and reliability of the content of each survey is depend on Macromill Carenet Inc verified or guaranteed. In addition, the participants were women in their 40 s to 80 s, with a mean age of 55.6 years. Of the 1,031 patients, approximately one-third were elderly and aged ≥ 60 years (mean age, 66.1 years). Moreover, because this survey was web-based, it cannot be ruled out that the participants may have been limited to a population of individuals or their family members capable of using the Internet. Despite these limitations, premenopausal women, who are assumed to have decreased estrogen levels, accounted for one-fourth (25.0%) of the participants. The fact that the study population was not solely represented by postmenopausal women strongly suggests that it may represent a target population for research into the essential features of GSM. Self-reporting for all answers, including whether or not menopause is present and its symptoms, is also considered a limitation in terms of accuracy. Furthermore, while similar studies in the past have typically included Westerners, this is the first report from Japan in Asia involving 10,000 participants, the largest study sample reported to date, thus providing appropriate information on the status of GSM among women in their 40 s and older. The largest limitation of the survey was that, in contrast to the questionnaire on treatment satisfaction showing satisfaction in as many as 60% of the respondents, 24% of the respondents reported having been untreated for their symptoms despite presenting to clinics, and only 1.5% or less than one-tenth of those who reported having received or currently receiving medical consultation (15.4% of all respondents) continued treatment at clinics, which was inconsistent with the fact that many reported having remained untreated despite medical consultation and very few continued with treatment, which thus suggests a limitation of the web-based questionnaire survey.

## Conclusions

This first nationwide web-based questionnaire survey provided insight into how women were coping with their symptoms associated with VVA and GSM in Japan.

The survey results suggest that GSM tends to become progressive and constitutes a health risk for affected women who are unwilling or fail to seek consultation about their symptoms. In addition, by definition, GSM crosses the boundary between gynecology (genital organs) and urology (lower urinary tract); as a result, affected women may have difficulty finding specialists well versed enough in both to address their symptoms, thus leading to underdiagnosis and undertreatment of GSM in these women. To complicate the matter, survey results suggest that limited appropriate treatment options are available even from the perspective of healthcare providers, that healthcare providers may be at a loss as to how to better address the needs of women with VVA and GSM, and that these women tend to receive far from satisfactory treatment, with one-fourth receiving no treatment and only one-tenth continuing with hospital-provided treatment.

Given the current circumstances, the most urgent mission of gynecologists and urologists, as primary physicians involved in the management of VVA and GSM, is to build awareness that VVA and GSM may be prevented in women at risk through highly specialized preemptive care and that the symptoms of GSM may also be ameliorated in affected women through highly specialized treatment. It is also expected that multifaceted, non-medical intervention termed “fem-tech” or “fem-care” may have a role as an adjunct to medical intervention in VVA and GSM. We further need a longitudinal study to follow the long-term prognosis of GSM patients seemed necessary although the current study was a cross-sectional study.

## Data Availability

The datasets generated and/or analyzed during the current study are not publicly available due to participant privacy, but they are available from the corresponding author on reasonable request.

## References

[1] Portman DJ, Gass ML, Vulvovaginal Atrophy Terminology Consensus Conference Panel (2014). Genitourinary syndrome of menopause: new terminology for vulvovaginal atrophy from the International Society for the Study of Women’s Sexual Health and the North American Menopause Society. Menopause.

[2] Portman DJ, Gass ML, Vulvovaginal Atrophy Terminology Consensus Conference Panel (2014). Genitourinary syndrome of menopause: new terminology for vulvovaginal atrophy from the International Society for the Study of Women’s Sexual Health and the North American Menopause Society. J Sex Med.

[3] Ohta H, Hatta M, Ota K, Yoshikata R, Salvatore S (2020). Online survey of genital and urinary symptoms among Japanese women aged between 40 and 90 years. Climacteric.

[4] Osuga Y, Okamura K, Ando F, Shimokata H (2013). Prevalence of lower urinary tract symptoms in middle-aged and elderly Japanese. Geriatr Gerontol Int.

[5] Gabes M, Knuttel H, Stute P, Christian J, Apfelbacher CJ (2019). Measurement properties of patient-reported outcome measures (PROMs) for women with genitourinary syndrome of menopause: a systematic review. Menopause.

[6] Barbieri RL (1992). Hormone treatment of endometriosis: the estrogen threshold hypothesis. Am J Obstet Gynecol.

[7] Cagnacci A, Gallo M, Gambacciani M, Lello S, SocietàItalianadellaMenopausa (SIM) and the SocietàItalianadellaTerzaEtà (SIGiTE) (2019). Joint recommendations for the diagnosis and treatment of volvo-vaginal atrophy in women in the peri- and post-menopausal phases from the Società Italiana della Menopausa (SIM) and the Società Italiana della Terza Età (SIGiTE). Minerva Ginecol.

[8] Santoro N, Komi J (2009). Prevalence and impact of vaginal symptoms among postmenopausal women. J Sex Med.

[9] Calleja-Agius J, Brincat MP (2015). The urogenital system and the menopause. Climacteric.

[10] Nappi RE, Palacios S (2014). Impact of vulvovaginal atrophy on sexual health and quality of life at postmenopause. Climacteric.

[11] Management of symptomatic vulvovaginal atrophy (2013). 2013 position statement of the North American Menopause Society. Menopause.

[12] DiBonaventura M, Luo X, Moffatt M, Bushmakin AG, Kumar M, Bobula J (2015). The association between vulvovaginal atrophy symptoms and quality of life among postmenopausal women in the United States and Western Europe. J Womens Health (Larchmt).

[13] Gandhi J, Chen A, Dagur G, Suh Y, Smith N, Cali B, Khan SA (2016). Genitourinary syndrome of menopause: an overview of clinical manifestations, pathophysiology, etiology, evaluation, and management. Am J Obstet Gynecol.

[14] Manonai J, Theppisai U, Suthutvoravut S, Udomsubpayakul U, Chittacharoen A (2001). The effect of estradiol vaginal tablet and conjugated estrogen cream on urogenital symptoms in postmenopausal women: a comparative study. J Obstet Gynaecol Res.

[15] Rioux JE, Devlin C, Gelfand MM, Steinberg WM, Hepburn DS (2000). 17 beta-estradiol vaginal tablet versus conjugated equine estrogen vaginal cream to relieve menopausal astrophic vaginitis. Menopause.

[16] Eriksen PS, Rasmussen H (1992). Low-dose 17 beta-estradiol vaginal tablets in the treatment of atrophic vaginitis: a double-blind placebo controlled study. Eur J Obstet Gynecol Reprod Biol.

[17] Garcia LE (1993). Efficiency of vaginal ovules of estriol for treatment of symptoms of menopause. Invest Med Int.

[18] Weisberg E, Ayton R, Darling G, Farrell E, Murkies A, O’Neil S, Kirkegard Y, Fraser S (2005). Endometrial and vaginal effects of low-dose estradiol delivered by vaginal ring or vaginal tablet. Climacteric.

[19] Labrie F, Archer DF, Kotun W, Vachon A, Young D, Frenette L, Portman D, Montesino M, Côté I, Parent J, Lavoie L, Martel C, Vaillancourt M, Balser J, Moyneur É, members of the VVA Prasterone Research Group (2016). Efficacy of intravaginal dehydroepiandrosterone (DHEA) on moderate to severe dyspareunia and vaginal dryness, symptoms of vulvovaginal atrophy, and of the genitourinary syndrome of menopause. Menopause.

[20] Portman DJ, Labrie F, Archer DF, Bouchard C, Cusan L, Girard G, Ayotte N, Koltun W, Blouin F, Young D, Wade A, Martel C, Dubé R, other participating members of VVA Prasterone Group (2015). Lack of effect of intravaginal dehydroepiandrosterone (DHEA, prasterone) on the endometrium in postmenopausal women. Menopause.

[21] Portman DJ, Bachmann GA, Simon JA, Ospemifene Study Group (2013). Ospemifene, a novel selective estrogen receptor modulator for treating dyspareunia associated with postmenopausal vulvar and vaginal atrophy. Menopause.

[22] Bachmann GA, Komi JO, Ospemifene Study Group (2010). Ospemifene effectively treats vulvovaginal atrophy in postmenopausal women: results from a pivotal phase 3 study. Menopause.

[23] Archer DF, Godstein SR, Simon JA, Waldbaum AS, Sussman SA, Altomare C, Zhu J, Yoshida Y, Schaffer S, Soulban G (2019). Efficacy and safety of ospemifene in postmenopausal women with moderate-to-severe vaginal dryness: a phase 3, randomized, double-blind, placebo-controlled, multicenter trial. Menopause.

[24] Pastore LW, Carter RA, Hulka BS, Wells E (2004). Self-reported urogenital symptoms in postmenopausal women: Women’s Health Initiative. Maturitas.

[25] Levine KB, Williams RE, Hartmann KE (2008). Vulvovaginal atrophy is strongly associated with female sexual dysfunction among sexually active postmenopausal women. Menopause.

[26] Palma F, Vope A, Villa P, Cagnacci A, Writing group of AGATA study (2016). Vaginal atrophy of women in postmenopause. Results from a multicentric observational study: AGTA study. Maturitas.

[27] Simon JA, Kokoto-Kierepa M, Goldstein J, Nappi RE (2013). Vaginal health in the United States: results from the Vaginal Health: Insights Views & Attitudes survey. Menopause.

[28] Kingsberg SA, Krychman M, Graham S, Bernick B, Mirkin S (2017). The Women’s EMPOWER Survey: identifying women’s perceptions on vulvar and vaginal atrophy and its treatment. J Sex Med.

[29] Kingsberg SA, Wysocki S, Magnus L, Krychman ML (2013). Vulvar and vaginal atrophy in postmenopausal women: findings from the REVIVE (Real Women’s Viewers of Treatment Options for Menopausal Vaginal Changes) survey. J Sex Med.

[30] Krychman M, Graham S, Bernick B, Mirkin S, Kingsberg SA (2017). The women’s EMPOWER survey: women’s knowledge and awareness of treatment options for vulvar and vaginal atrophy remains inadequate. J Sex Med.

[31] Nappi RE, Palacios S, Bruyniks N, Particco M, Panay N, EVES Study Investigators (2019). The burden of vulvovaginal atrophy on women’s daily living: implications on quality of life from a face-to-face real-life survey. Menopause.

[32] Shifren JL, Zincavage R, Cho EL, Magnavita A, Portman DJ, Krychman ML, Simon JA, Kingsberg SA, Rosen RC (2019). Women’s experience of vulvovaginal symptoms associated with menopause. Menopause.

[33] World Health Organization: Global status report on noncommunicable diseases 2014. Geneva (Switzerland): WHO [cited 2019 May 21]. Available from: https://www.who.int/nmh/pulications/ncd-status-report-2014/en/.

